# VPS33B and VIPAR are essential for epidermal lamellar body biogenesis and function

**DOI:** 10.1016/j.bbadis.2018.01.028

**Published:** 2018-05

**Authors:** Clare Rogerson, Paul Gissen

**Affiliations:** a MRC Laboratory for Molecular Cell Biology, University College London, London WC1E 6BT, UK; b Institute of Child Health, University College London, London WC1N 1EH, UK; c Inherited Metabolic Diseases Unit, Great Ormond Street Hospital, London WC1N 3JH, UK

**Keywords:** ARC, Arthrogryposis Renal dysfunction and Cholestasis, ARKID, Autosomal Recessive Keratoderma-Ichthyosis-Deafness, CORVET, core vacuole/endosome tethering, D, dermis, H&E, hematoxylin and eosin, HOPS, homotypic fusion and vacuole protein sorting, LB, lamellar body, SB, stratum basale, SC, stratum corneum, SG, stratum granulosum, SS, stratum spinosum, TEER, trans-epithelial electrical resistance, TEM, transmission electron microscopy, TEWL, trans-epithelial water loss, ARC syndrome, ARKID syndrome, CHEVI complex, Lamellar bodies, VIPAR, VPS33B

## Abstract

Mutations in *VPS33B* and *VIPAS39* cause the severe multisystem disorder *A*rthrogryposis, *R*enal dysfunction and *C*holestasis (ARC) syndrome. Amongst other symptoms, patients with ARC syndrome suffer from severe ichthyosis. Roles for VPS33B and VIPAR have been reported in lysosome-related organelle biogenesis, integrin recycling, collagen homeostasis and maintenance of cell polarity. Mouse knockouts of *Vps33b* or *Vipas39* are good models of ARC syndrome and develop an ichthyotic phenotype. We demonstrate that the skin manifestations in Vps33b and Vipar deficient mice are histologically similar to those of patients with ARC syndrome. Histological, immunofluorescent and electron microscopic analysis of Vps33b and Vipar deficient mouse skin biopsies and isolated primary cells showed that epidermal lamellar bodies, which are essential for skin barrier function, had abnormal morphology and the localisation of lamellar body cargo was disrupted. Stratum corneum formation was affected, with increased corneocyte thickness, decreased thickness of the cornified envelope and reduced deposition of lipids. These defects impact epidermal homeostasis and lead to abnormal barrier formation causing the skin phenotype in Vps33b and Vipar deficient mice and patients with ARC syndrome.

## Highlights

• Vps33b and Vipar deficient mice are good models of ARC syndrome skin disease.
• Epidermal differentiation is disrupted in Vps33b and Vipar deficient murine skin.
• Vps33b and Vipar deficient dry skin is linked to abnormal lamellar body biogenesis.

## 1 Introduction

Patients with mutations in the genes *VPS33B* and *VIPAS39* have been reported with *A*rthrogryposis, *R*enal dysfunction and *C*holestasis (ARC) syndrome (OMIM #208085 and #613404) [1,2]. ARC syndrome is a rare autosomal recessive multisystem disorder and patients characteristically present with arthrogryposis, renal proximal tubule dysfunction, neonatal cholestasis and severe failure to thrive [3]. Patients also present with other symptoms as part of this syndrome including ichthyosis, sensorineural deafness, abnormal platelet α-granule biosynthesis, osteopenia, absent corpus callosum, recurrent infections and mild dysmorphia [3]. The majority of those affected by ARC syndrome die in infancy, generally from sepsis, dehydration, or acidosis caused by recurrent infections [4], however, a few patients have been reported to survive until childhood with an attenuated ARC syndrome phenotype [5,6].

We recently described *A*utosomal *R*ecessive *K*eratoderma *I*chthyosis *D*eafness (ARKID) syndrome, a syndrome allelic to ARC syndrome, caused by a novel missense mutation in *VPS33B*: c.[392G > A;390G > A] p.Gly131Glu, patients with ARKID syndrome also present with symptoms including severe palmoplantar hyperkeratosis [7]. Although patients with VPS33B and VIPAR deficiencies develop dry scaly skin conditions both as part of ARC syndrome [3] or the newly identified ARKID syndrome [7], the underlying cause of the skin disease has not yet been determined.

The uppermost layer of the skin is a highly organised stratified epithelium formed of four distinct layers of keratinocytes. In the basal layer, the stratum basale (SB), keratinocytes proliferate and their daughter cells populate the upper layers. These cells differentiate into cells of the spinous layer, the stratum spinosum (SS), cells of the granular layer, the stratum granulosum (SG), and then into cells of the uppermost layer, the stratum corneum (SC), losing their organelle contents and becoming progressively cornified as they differentiate into enucleate corneocytes [8]. Formation of the SC is heavily dependent on lamellar bodies (LBs), lysosome-related organelles produced in SG keratinocytes and secreted at the junction between the SG and SC. Amongst other cargo LBs contain lipids and lipid modifying enzymes which contribute to the formation and homeostasis of the SC [9].

Previous analysis of the ultrastructure of skin from patients with ARC syndrome identified unexpected lipid bilayer structures and inclusions in the SC of patients with a *VPS33B* mutation: c.701-1G > C p.Asp234His, which leads to truncation of the VPS33B protein [10]. These structures were not present in the SC of healthy human controls and Hershkovitz et al. attributed the abnormal lamellar structures in the SC to the inability of LBs to secrete their contents at the SG-SC junction, leading to “entombed lamellar granules” [10]. Biopsies from patients with ARKID syndrome and mice deficient for Vps33b also demonstrated abnormal LB structures in the SG and retention of lipid layers in the SC, which further indicates defects in LB function in VPS33B deficiency [7].

The proteins encoded by the genes *VPS33B* and *VIPAS39*, VPS33B and VIPAR respectively, interact and are hypothesized to act as regulators of intracellular vesicular fusion events [2,11,12]. VPS33B and VIPAR proteins show a high degree of similarity to their yeast homologues Vps33p and Vps16p, which are required for protein trafficking to the yeast vacuole [13] as components of the class c core vacuole/endosome tethering (CORVET) and homotypic fusion and vacuole protein sorting (HOPS) multiprotein complexes [14]. Metazoans have two other homologues of Vps33p and Vps16p: VPS33A and VPS16A. VPS33A and VPS16A interact and act as components of the mammalian HOPS and CORVET complexes [[15], [16], [17]] whilst VPS33B and VIPAR are thought to act as a component of a distinct and as yet only partly characterised tethering complex, the class C homologues in endosome-vesicle interaction (CHEVI) complex [11,12].

The epidermal defects of patients with ARC and ARKID syndromes could be a direct effect of deficient VPS33B-VIPAR trafficking; in LB biogenesis and secretion a tethering complex could be important for delivery of cargo proteins to LBs or important for tethering and secretion of LBs at the plasma membrane. As keratinocytes in the SG differentiate they secrete LBs at their apical surface, at the interface with the SC [18], and as the keratinocytes differentiate into corneocytes of the SC their intracellular structures are degraded through release of lysosomal enzymes [8]. The “entombed” LBs described by Hershkovitz et al. [10], could therefore be due to deficiency of the VPS33B-VIPAR tethering complex in secretion of LB contents, in the biogenesis of LBs, which could affect their secretion, or could also be caused by defects in degradation of intracellular organelles.

The VPS33B-VIPAR complex has previously been reported to have diverse functions, from α-granule formation in megakaryocytes [5,19], platelet integrin recycling [20], delivery of a collagen modifying enzyme, LH3, to collagen in kidney cells and fibroblasts [21], Rab11a-dependent apical protein targeting in hepatocytes [2,22] to exosome maturation [23]. Therefore, the epidermal defects of patients with ARC and ARKID syndromes could be an indirect effect of defects in previously described VPS33B-VIPAR complex functions, for example, epidermal defects may be secondary to alterations in LH3-dependent collagen modification, leading to altered basal lamina structure or alterations in apical protein targeting. This could affect differentiation and downstream signalling, or establishment of polarity that could impact LB biogenesis and secretion.

Several groups have generated murine models to better understand the pathophysiology of ARC syndrome, however, constitutive and early embryonic Vps33b knockout is lethal [5,19]. We generated tamoxifen-inducible ubiquitous Vps33b and Vipar knockout mice: *Vps33b*^*fl/fl*^*ER*^*T2*^ and *Vipas39*^*fl/fl*^*ER*^*T2*^ mice and induce knockout 4–5 weeks after birth [5,21]. The mice do not develop some phenotypes associated with ARC syndrome, such as visible joint contractures or severe renal dysfunction, possibly due to induction of the knockout post-development. However, both *Vps33b*^*fl/fl*^*ER*^*T2*^ and *Vipas39*^*fl/fl*^*ER*^*T2*^ mice develop dry scaly skin, and alterations in LB and SC ultrastructure have been reported in *Vps33b*^*fl/fl*^*ER*^*T2*^ epidermis [3,5,21]. How VPS33B and VIPAR deficiency affects epidermal homeostasis and LB secretion, has yet to be determined. In order to better understand the underlying causative mechanisms of the dry skin phenotype in patients with ARC and ARKID syndromes, we analysed the histology of the skin in our Vps33b and Vipar deficient mice and investigated the effects of VPS33B and VIPAR deficiency on the function of epidermal junctions, structure of dermal collagen and function of epidermal LBs.

## 2 Materials and methods

### 2.1 Antibodies and reagents

The following primary antibodies were used: anti-β-catenin (ab2365, Abcam), anti-claudin-1 (717800, ThermoFisher), anti-cleaved caspase-3 (9661S Cell Signalling), anti-collagen I (NB600-408, Novus Biologicals), anti-E-cadherin (610181, BD Biosciences), anti-K6A (905701, Biolegend), anti-K10 (905401, Biolegend), anti-K14 (ab7800, Abcam), anti-Ki67 (ab15580, Abcam) and anti-KLK5 (MAB7236, R&D Systems). All fluorescent secondary antibodies were Alexa Fluor conjugates (Life Technologies, UK). All reagents were from Sigma-Aldrich unless stated.

### 2.2 *Vps33b*^*fl/fl*^*-ER*^*T2*^ and *Vipas39*^*fl/fl*^*-ER*^*T2*^ mice and trans-epidermal water loss (TEWL) measurements

Tamoxifen induction of the exon knockouts in *Vps33b*^*fl/fl*^*-ER*^*T2*^ and *Vipas39*^*fl/fl*^*-ER*^*T2*^ mice have been described previously [5,7,21]. Control mice are either *Vps33b*^*+/+*^ or *Vipas39*^*+/+*^; tamoxifen injection did not cause a skin phenotype and therefore the control mice were not injected with tamoxifen. Where control mice are indicated results from *Vps33b*^*+/+*^ and *Vipas39*^*+/+*^ mice were pooled due to invariability of results. United Kingdom Home Office approval was obtained for all experiments, licence number PPL 70/7470, in accordance with the Animals (Scientific Procedures) Act of 1986. Photos were obtained with the permission of the Named Veterinary Surgeon of the Biological Services Unit of UCL. TEWL measurements across murine ventral abdomen were performed with a VapoMeter (Delfin Technologies) according to the manufacturer's instructions; fur was cut short with an electric hair trimmer immediately prior to the readings and the average of three measurements per mouse was used.

### 2.3 Primary murine keratinocyte isolation, maintenance and stratification

In a method adapted from Lichti et al. [24] primary murine keratinocytes were isolated from control, *Vps33b*^*fl/fl*^*-ER*^*T2*^ and *Vipas39*^*fl/fl*^*-ER*^*T2*^ tail skin. The final cell suspension was diluted to 2.5 × 10^5^ cells/ml for control cells and 3 × 10^5^ cells/ml for *Vps33b*^*fl/fl*^*-ER*^*T2*^ and *Vipas39*^*fl/fl*^*-ER*^*T2*^ cells for seeding on fibronectin coated dishes. Cells were grown at 37 °C, 5% CO_2_ in a humidified atmosphere. Primary murine keratinocytes were stratified with CnT-Prime-3D (CELLnTEC) according to the manufacturer's protocol: keratinocytes at 5 × 10^5^ cells/ml in CnT-Prime (CELLnTEC) were seeded in polyester 0.4 μm Transwell inserts (Corning), at confluency, CnT-Prime was replaced with CnT-Prime-3D and the following day the insert raised to the air-liquid interface and cultured for 12 days.

### 2.4 Trans-epithelial electrical resistance measurements of stratified primary keratinocyte cultures

Fresh media was added outside and inside Transwell inserts containing stratified keratinocyte cultures, as described in Section 2.2, and equilibrated for 30 min. Readings were taken with a SSTX01 electrode (Millipore) connected to a Millicell ERS-2 Voltohmmeter (Millipore) according to the manufacturer's protocol. Resistance was calculated as raw resistance subtracted by the average resistance of two empty inserts. Duplicate well measurements were averaged. The Unit Area Resistance (Ωcm^2^) was calculated as the product of the resistance and surface area of the inserts.

### 2.5 Primary murine fibroblast isolation and culture

Fibroblasts were isolated from dermal samples generated during keratinocyte isolation, see Section 2.2, which were minced and incubated with 1×trypsin-EDTA at 37 °C with 5% CO_2_ for 20 min. Approximately half the cell suspension was removed and trypsin-EDTA inactivated in fibroblast medium (DMEM high glucose with l-glutamine, 15% FBS, 1× penicillin-streptomycin and 0.1 mM β-mercaptoethanol). Fresh trypsin was added and the suspension returned to the incubator. This was repeated 3–5 times. Remaining suspension was collected through a 100 μm cell strainer and the suspension centrifuged twice at 200*g* for 5 min. The pellet was resuspended and plated into suitable culture dishes.

### 2.6 Secreted extracellular matrix preparation from primary murine fibroblasts

In a method adapted from Caley et al. [25] confluent primary murine fibroblasts were cultured with the addition of 100 μM ascorbic acid for 10 days. The slides were decellularised and stained for collagen I. The ImageJ Directionality plugin measured fibre alignment [26].

### 2.7 Wound healing assays

Confluent primary murine fibroblasts were scratched with a 200 μl pipette tip, held at 90° to the well surface. An EVOS XL Core widefield light microscope (Life Technologies) was used to image the scratch at 0, 16 and 24 h. The area of the scratch was quantified using the BoneJ ImageJ plugin [27].

### 2.8 Cell spreading assays

Primary murine keratinocytes or fibroblasts at 1 × 10^5^ cells/ml were seeded into tissue cultured treated dishes, fixed after 1 h and stained with Alexa-labelled wheat germ agglutinin and DAPI. Three images per experiment were analysed with CellProfiler software [28].

### 2.9 Co-culture of primary murine keratinocytes and fibroblasts for a full thickness skin model

Primary murine fibroblasts cultured in CnT-Prime Fibroblast (CELLnTEC) were plated at 1 × 10^5^ cells/ml into 0.4 μm pore Millicell hanging cell culture inserts (Millipore). On day 9 or 10, CnT-Prime Fibroblast was removed and primary murine keratinocytes were seeded at 2 × 10^5^ cells/ml in CnT-Prime-FTAL (CELLnTEC). After three days the inserts were lifted to the air-liquid interface and the models cultured for 12 days.

### 2.10 Fixation, processing and histological staining of murine epidermal tissue samples

Murine epidermal skin samples were frozen in optimum cutting temperature compound in an isopentane bath cooled over liquid nitrogen or fixed in 10% formalin. Formalin fixed samples were paraffin embedded, sectioned and processed for histological staining by the Biomedical Research Centre at the Institute of Child Health, London, UK. Hematoxylin and eosin (H&E) and picrosirius red stains were imaged on a Zeiss Axioplan2 microscope with a Zeiss AxioCam HRc colour camera. Epidermal thickness was calculated using the BoneJ ImageJ plugin [27]. The number of cells was counted using the ImageJ Cell Counter plugin and reported per 100 μm of the dermal-epidermal junction. Keratinocyte size was calculated as total epidermal area divided by the number of keratinocyte nuclei.

### 2.11 Immunohistochemistry

Slides with paraffin embedded sections were stained as described previously [22] and images taken with a Zeiss Axioplan2 fitted with a Zeiss Axiocam HRc colour camera.

### 2.12 Immunofluorescent staining

Cryosections were fixed in 4% PFA and −20 °C methanol, cell or matrix cultures were fixed in 4% PFA. Sections were quenched with 50 mM ammonium chloride and permeabilised with 0.25% Triton X-100. Blocking was performed with 1% goat or donkey serum in PBS with 0.5% Triton X-100. Primary antibodies were diluted in blocking solution and incubated overnight at 4 °C. Alexa-conjugated secondary antibodies, also diluted in blocking solution, were incubated with samples for 2 h at RT. Samples were counterstained with DAPI, mounted with Prolong Gold (Life Technologies) and imaged on a Leica TCS SP5 confocal microscope. Ki67 positive nuclei were counted using the ImageJ Cell Counter plugin.

### 2.13 Oil red O (ORO) staining

Cryosections were fixed in 4% PFA, washed with tap water and incubated with 60% isopropanol for 5 min. A 3 mg/ml stock solution of ORO in 100% isopropanol was diluted 3:2 in deionised water and filtered. Slides were incubated with ORO solution for 15 min and rinsed with tap water. Slides were counterstained with DAPI mounted in Prolong Gold and imaged on a Leica TCS SP5 confocal microscope.

### 2.14 Transmission electron microscopy (TEM)

1.5 mm mouse skin biopsies, fixed in 4% glutaraldehyde (TAAB) in 0.1 M sodium phosphate, were prepared and imaged as described previously [7].

### 2.15 Statistics

All analyses were performed using GraphPad Prism 7. For the comparison of two datasets, two-tailed unpaired *t*-tests were performed and an F-test used to ensure that the variances were not significantly different between the two datasets. To compare three or more data sets an ordinary one-way ANOVA was performed and a Brown-Forsythe test was used to ensure the variances were not significantly different between the datasets. A Tukey's multiple comparisons post-test was then used to analyse differences between pairs of datasets. For analysis in Fig. 2D, a non-parametric Kruskal-Wallis test was performed, with a Dunn's multiple comparisons post-test.

## 3 Results

### 3.1 Vps33b and Vipar deficient mice have a dry skin phenotype with defective water barrier function

**Fig. 1:**
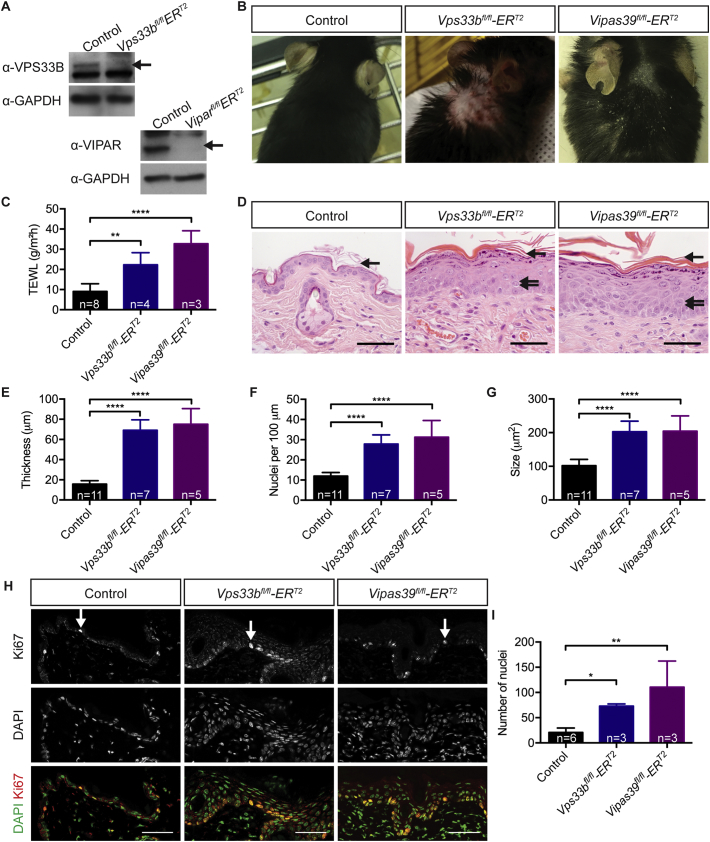
Vps33b and Vipar deficient mice develop dry skin with a defective water loss barrier that is characterised by hyperplasia and hyperproliferation. A - Control, *Vps33b*^*fl/fl*^*ER*^*T2*^ and *Vipas39*^*fl/fl*^*ER*^*T2*^ keratinocyte lysates blotted for VPS33B, VIPAR and GAPDH expression, arrows indicate VPS33B and VIPAR specific bands. B - Photos of dorsal skin of control, *Vps33b*^*fl/fl*^*ER*^*T2*^ and *Vipas39*^*fl/fl*^*ER*^*T2*^ mice. *Vps33b*^*fl/fl*^*ER*^*T2*^ photo reproduced from [5]. C - Trans-epidermal water loss (TEWL) of control, *Vps33b*^*fl/fl*^*ER*^*T2*^ and *Vipas39*^*fl/fl*^*ER*^*T2*^ mice. D - H&E staining of control, *Vps33b*^*fl/fl*^*ER*^*T2*^ and *Vipas39*^*fl/fl*^*ER*^*T2*^ skin with increased purple granular staining, spongiosis (double arrows) and thicker SC (single arrow). E - Epidermal thickness of control, *Vps33b*^*fl/fl*^*ER*^*T2*^ and *Vipas39*^*fl/fl*^*ER*^*T2*^ mice, the distance from the dermal-epidermal junction to the SG-SC junction. F - The number of nuclei in the epidermis of control, *Vps33b*^*fl/fl*^*ER*^*T2*^ and *Vipas39*^*fl/fl*^*ER*^*T2*^ mice. G - Average keratinocyte size of control, *Vps33b*^*fl/fl*^*ER*^*T2*^ and *Vipas39*^*fl/fl*^*ER*^*T2*^ mice: epidermal area divided by number of nuclei. H - Representative Ki67 staining of control, *Vps33b*^*fl/fl*^*ER*^*T2*^ and *Vipas39*^*fl/fl*^*ER*^*T2*^ skin sections, counterstained with DAPI. I - Quantification of the number of Ki67 stained nuclei per mm of epidermis. Western blots are representative of two independent western blotting experiments. Scale bars = 50 μm. Immunofluorescent images are a maximum projection of a z-stack. For epidermal measurements three fields of view were measured per mouse, the number of independent murine samples mice analysed (n) are indicated on the graphs. * *p* ≤ 0.05, ** *p* ≤ 0.01, **** *p* ≤ 0.0001.

Three to four weeks after induction of the knockout both *Vps33b*^*fl/fl*^*ER*^*T2*^ and *Vipas39*^*fl/fl*^*ER*^*T2*^ mice lose VPS33B or VIPAR protein expression in epidermal keratinocytes, Fig. 1A, and develop a visible dry flaky skin phenotype, Fig. 1B, with increased water loss, Fig. 1C, indicating a disrupted barrier to water loss in Vps33b and Vipar deficient mice.

The epidermis of *Vps33b*^*fl/fl*^*ER*^*T2*^ and *Vipas39*^*fl/fl*^*ER*^*T2*^ mice is thicker than control epidermis, with increased thickness of the SB and SS (acanthosis), the SG (hypergranulosis) and the SC (hyperkeratosis), Fig. 1D, as previously reported in *Vps33b*^*fl/fl*^*ER*^*T2*^ mice [7]. The epidermal height increased from an average of 15.82 μm in controls to 69.13 and 75.04 μm in *Vps33b*^*fl/fl*^*ER*^*T2*^ and *Vipas39*^*fl/fl*^*ER*^*T2*^ mice respectively, Fig. 1E. This is influenced by an increase in keratinocyte number (hyperplasia), from approximately 11 keratinocytes per 100 μm to 28 and 31 keratinocytes per 100 μm, Fig. 1F, and an increase in keratinocyte size, Fig. 1G. Control keratinocytes had an average size of 101.8 μm^2^, consistent with previously reported figures [29,30], but *Vps33b*^*fl/fl*^*ER*^*T2*^ and *Vipas39*^*fl/fl*^*ER*^*T2*^ keratinocytes were significantly larger with average areas of 202.9 and 204.6 μm^2^ respectively. There was an increase in dark purple punctate “granular” staining in the upper SG, indicating changes in the structure of SG cells, and gaps between cells (spongiosis) in the SG and SS, Fig. 1D and Supplementary Fig. 1A, which is a phenomenon previously noted to be associated with the response of the epidermis to decreased barrier function [31,32].

Proliferation was increased in both Vps33b and Vipar deficient mice compared to control; there was an increase in the number of nuclei expressing the proliferation marker Ki67, Fig. 1H and I, and increased expression of hyperproliferation-associated keratin 6A (K6A) in *Vps33b*^*fl/fl*^*ER*^*T2*^ and *Vipas39*^*fl/fl*^*ER*^*T2*^ epidermis, Supplementary Fig. 1B. There was no difference identified in the expression of the apoptotic marker cleaved caspase-3, Supplementary Fig. 1C, suggesting epidermal hyperplasia in Vps33b and Vipar deficient mice is due to increased keratinocyte proliferation.

### 3.2 Epidermal differentiation is affected in Vps33b and Vipar deficient mice

**Fig. 2:**
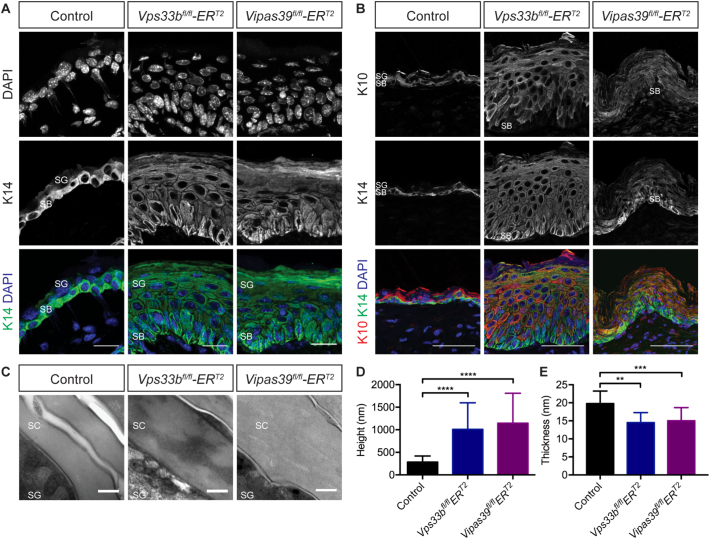
Vps33b and Vipar deficient mice have defects in terminal keratinocyte differentiation. A - K14 staining in control, *Vps33b*^*fl/fl*^*ER*^*T2*^ and *Vipas39*^*fl/fl*^*ER*^*T2*^ skin sections, counterstained with DAPI. Scale bars = 20 μm. Images are a maximum projection of a z-stack and are representative of results from at least six control and three *Vps33b*^*fl/fl*^*ER*^*T2*^ and *Vipas39*^*fl/fl*^*ER*^*T2*^ independent murine biopsies. B - TEM images of control, *Vps33b*^*fl/fl*^*ER*^*T2*^ and *Vipas39*^*fl/fl*^*ER*^*T2*^ SC. Scale bars = 200 nm. Images are representative of at least two independent murine biopsies per genotype. C - Quantification of corneocyte height: the two corneocytes closest to the SG-SC junction were measured. D - Quantification of cornified envelope thickness. SB, SG and SC layers are indicated. ** *p* ≤ 0.01, *** *p* ≤ 0.001, ****p ≤ 0.0001.

All layers of the epidermis are formed in *Vps33b*^*fl/fl*^*ER*^*T2*^ and *Vipas39*^*fl/fl*^*ER*^*T2*^ epidermis, Fig. 1D, indicating that keratinocyte differentiation is occurring. However, the SG showed increased granular staining and the SC was thicker in appearance, suggesting defects in keratinocyte differentiation impacting cells of the SG and SC. In healthy epidermis Fig. 2, keratin 14 (K14) is highly expressed in basal keratinocytes and downregulated in the keratinocytes of the SS and SG [33], Fig. 2A, and as K14 expression is downregulated in differentiating keratinocytes the expression of K10 is upregulated [33], Fig. 2B. However, in both *Vps33b*^*fl/fl*^*ER*^*T2*^ and *Vipas39*^*fl/fl*^*ER*^*T2*^ epidermis K14 is not downregulated, Fig. 2A, although K10 is upregulated in suprabasal keratinocytes, Fig. 2B. This indicates there are no defects in the upregulation of expression of K10 but there may be other defects preventing K14 downregulation in Vps33b and Vipar deficient mice.

Keratinocyte differentiation culminates in the formation of the SC, a cornified layer consisting of enucleate corneocytes and intercellular lipid lamellae [8]. Control corneocytes are compact, devoid of cellular organelles and surrounded by a cornified envelope, Fig. 2C. In *Vps33b*^*fl/fl*^*ER*^*T2*^ and *Vipas39*^*fl/fl*^*ER*^*T2*^ epidermis the corneocytes are larger, Fig. 2D, and contain lipid-like layers and other diverse structures, suggesting defective keratinocyte terminal differentiation. *Vps33b*^*fl/fl*^*ER*^*T2*^ and *Vipas39*^*fl/fl*^*ER*^*T2*^ corneocytes also have thinner cornified envelopes, at an average of 14.53 nm and 15.03 nm respectively, compared to control corneocytes at 19.8 nm, Fig. 2E, further supporting the conclusion that there is a defect in terminal differentiation in Vps33b and Vipar deficient mice.

### 3.3 Epidermal junctions are unaffected in Vps33b and Vipar deficient mice

Increased water loss across Vps33b and Vipar deficient murine skin indicates the water barrier function of the epidermis is defective in *Vps33b*^*fl/fl*^*ER*^*T2*^ and *Vipas39*^*fl/fl*^*ER*^*T2*^ mice, Fig. 1C. The epidermal water barrier is made up of both epidermal junctions and SC lipid layers [34,35]. VPS33B and VIPAR deficiencies affect polarisation and formation of tight junctions in epithelial cells [2,22], which suggests the water barrier defect in *Vps33b*^*fl/fl*^*ER*^*T2*^ and *Vipas39*^*fl/fl*^*ER*^*T2*^ mice may be caused by defective junction formation, and defects in junction formation can lead to barrier disruption [[36], [37], [38]].

**Fig. 3:**
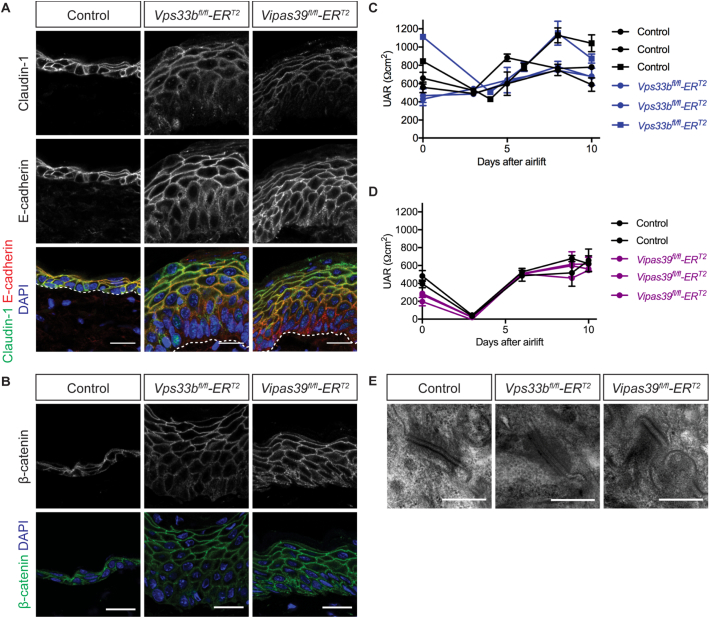
Vps33b and Vipar deficient skin disease is not caused by defects in epidermal junctions. A - Claudin-1 and E-cadherin staining in control, *Vps33b*^*fl/fl*^*ER*^*T2*^ and *Vipas39*^*fl/fl*^*ER*^*T2*^ skin sections, counterstained with DAPI. Scale bars = 20 μm. Dermo-epidermal junction (dashed line). B - β-catenin staining in control, *Vps33b*^*fl/fl*^*ER*^*T2*^ and *Vipas39*^*fl/fl*^*ER*^*T2*^ skin sections counterstained with DAPI. Scale bars = 20 μm. Immunofluorescent images are single z-slices from a z-stack and are representative of results from at least six control and three *Vps33b*^*fl/fl*^*ER*^*T2*^ and *Vipas39*^*fl/fl*^*ER*^*T2*^ independent murine biopsies. C - TEER readings of control and *Vps33b*^*fl/fl*^*ER*^*T2*^ keratinocyte cultures. Results from two experiments are shown with different symbols. D - TEER readings of individual control *Vipas39*^*fl/fl*^*ER*^*T2*^ keratinocyte cultures. E - TEM images of desmosomes between adjacent keratinocytes in control, *Vps33b*^*fl/fl*^*ER*^*T2*^ and *Vipas39*^*fl/fl*^*ER*^*T2*^ epidermis. Scale bars = 400 nm. TEM images are representative of at least two independent murine biopsies per genotype.

However, the tight junction protein claudin-1 and the adherens junction proteins E-cadherin and β-catenin in *Vps33b*^*fl/fl*^*ER*^*T2*^ and *Vipas39*^*fl/fl*^*ER*^*T2*^ epidermis localised to the plasma membrane, suggesting that the junctions are forming correctly in *Vps33b*^*fl/fl*^*ER*^*T2*^ and *Vipas39*^*fl/fl*^*ER*^*T2*^ mice, Fig. 3A and B. Tight junction function was confirmed *in vitro*; trans-epithelial electrical resistance (TEER) across stratified keratinocyte layers were not different to control keratinocytes, Fig. 3C and D, indicating the plasma membrane localised junctional proteins are forming functional tight junctions.

Desmosomes, important for cell-cell adhesion and mechanical strength in the epidermis also showed no overt change in ultrastructure in *Vps33b*^*fl/fl*^*ER*^*T2*^ and *Vipas39*^*fl/fl*^*ER*^*T2*^ epidermis, Fig. 3E. Further indicating defects in the water barrier function of the epidermis in Vps33b and Vipar deficient mice are unlikely to be due to alterations in epidermal junctions.

### 3.4 Vps33b and Vipar deficient mice display subtle defects in basement membrane formation and deposition of collagen I *in vitro*

We have recently shown that a collagen modifying enzyme, LH3, is mislocalised in VPS33B and VIPAR deficient mIMCD3 cells [21]. Altered LH3 distribution was also observed in fibroblasts from patients with the p.Gly131Glu novel VPS33B mutation in ARKID syndrome, with abnormal post-translational modification of collagen lysine residues and deficiencies in the basement membrane of the dermo-epidermal junction [7]. Knockout of LH3 in mice causes basement membrane abnormalities [39] and it is possible that disruption in LH3 delivery could cause the epidermal defects in Vps33b and Vipar deficient mice, and patients with ARC syndrome, by disrupting extracellular matrix formation, through deficiency of LH3-dependent collagen modifications.

**Fig. 4:**
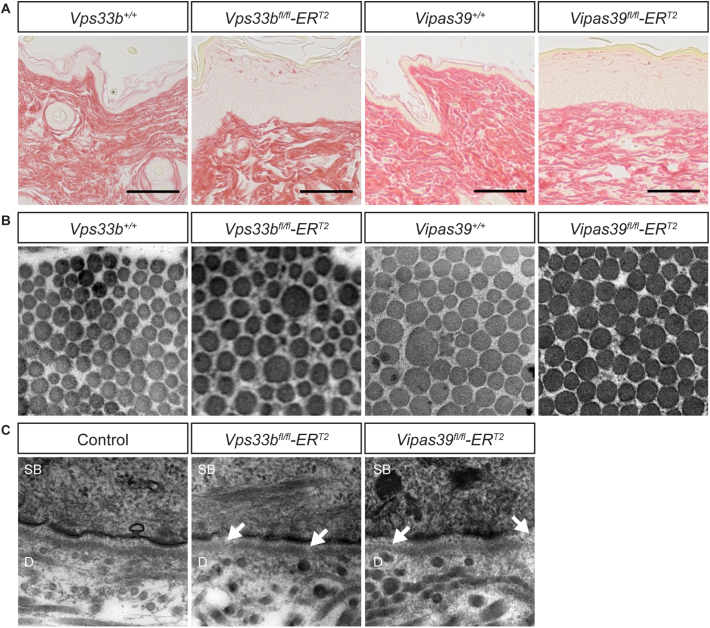
- Vps33b and Vipar deficient murine skin does not show gross changes in the morphology of dermal collagen fibres but areas of thin basement membrane suggest subtle defects in collagen structure. A - Picrosirius red staining of control (*Vps33b*^*+/+*^ and *Vipas39*^*+/+*^), *Vps33b*^*fl/fl*^*ER*^*T2*^ and *Vipas39*^*fl/fl*^*ER*^*T2*^ skin. In picrosirius red images collagen stains red. Images are representative of results from three independent murine biopsies per genotype. B - TEM images of collagen fibrils in control, *Vps33b*^*fl/fl*^*ER*^*T2*^ and *Vipas39*^*fl/fl*^*ER*^*T2*^ dermis. All images are 0.8 μm^2^. Images are representative of at least two independent murine biopsies per genotype. C - TEM images of the dermo-epidermal junction in control, *Vps33b*^*fl/fl*^*ER*^*T2*^ and *Vipas39*^*fl/fl*^*ER*^*T2*^ skin. Areas of thinner basement membrane (arrow). All images are 1 μm^2^. SB and dermis (D) are indicated. Images are representative of at least two independent murine biopsies per genotype.

There were no gross changes in the morphology of collagen and other fibres of the dermis in picrosirius red stained sections of control, *Vps33b*^*fl/fl*^*ER*^*T2*^ and *Vipas39*^*fl/fl*^*ER*^*T2*^ skin, Fig. 4A. *Vps33b*^*fl/fl*^*ER*^*T2*^ and *Vipas39*^*fl/fl*^*ER*^*T2*^ dermal collagen fibril size were also similar to those in control mice, Fig. 4B. However, electron microscopy of the dermo-epidermal junction showed areas of basement membrane that appeared thinner in *Vps33b*^*fl/fl*^*ER*^*T2*^ and *Vipas39*^*fl/fl*^*ER*^*T2*^ mice than in control mice indicating defects in deposition or organisation of the basement membrane, Fig. 4C, consistent with affected LH3 function.

**Fig. 5:**
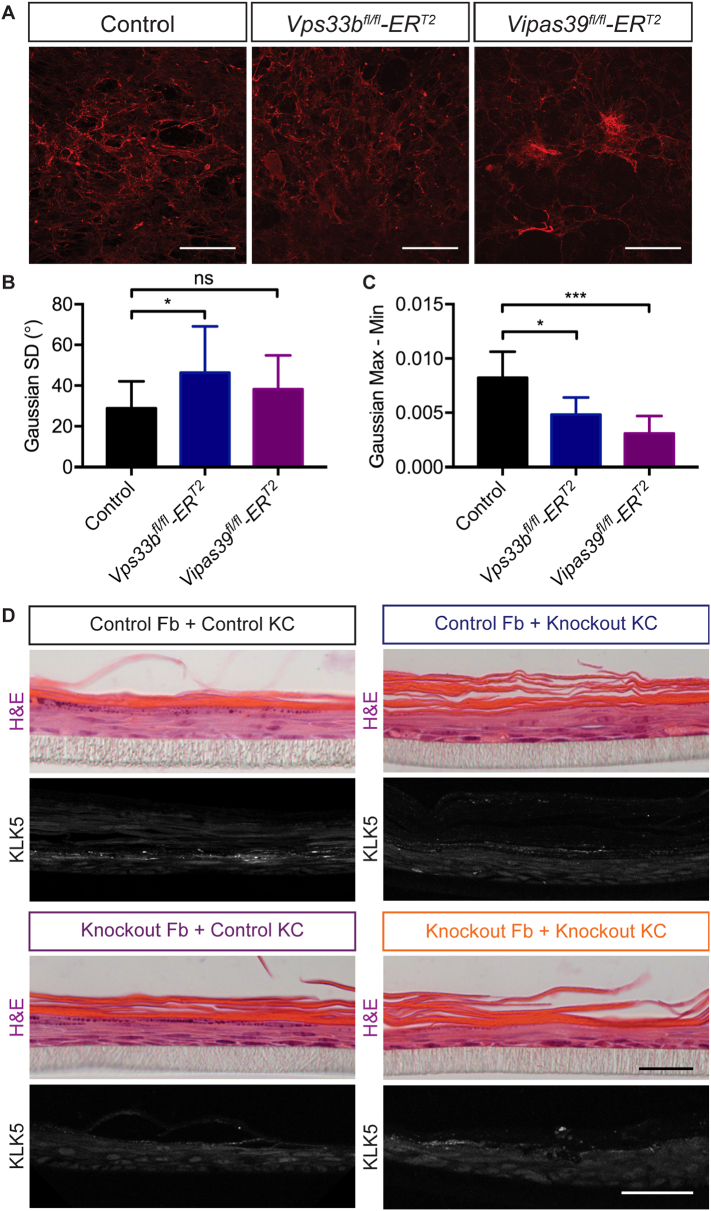
Vps33b and Vipar knockout affects collagen I deposition in fibroblasts and fibroblast function. A - Decellularised matrix from primary murine fibroblast cultures stained for collagen I. Scale bars = 200 μm. Images are maximum projections of a z-stack. Three images of two independent matrices analysed per genotype. B – Standard deviation (SD) of the Gaussian function of Directionality analysis of fibres in “A”. C - Range (Max - Min) of the Gaussian function of Directionality analysis of fibres in ‘A’. ns *p* > 0.05, * *p* ≤ 0.05, **** *p* ≤ 0.0001. D - Full thickness skin models of control or *Vipas39*^*fl/fl*^*ER*^*T2*^ primary fibroblasts with control or *Vps33b*^*fl/fl*^*ER*^*T2*^ primary keratinocytes stained with H&E, and anti-KLK5. H&E scale bar = 20 μm, KLK5 scale bar = 50 μm. Images are representative of at least three full thickness models.

Primary murine fibroblasts isolated from *Vps33b*^*fl/fl*^*ER*^*T2*^ and *Vipas39*^*fl/fl*^*ER*^*T2*^ mice secreted a more disorganised matrix *in vitro*, Fig. 5A. The ‘directionality’ of matrices, or the tendency of the fibres to align, was higher in control cultures, producing a tighter Gaussian distribution; the standard deviation of the Gaussian function was significantly higher in *Vps33b*^*fl/fl*^*ER*^*T2*^ and *Vipas39*^*fl/fl*^*ER*^*T2*^ fibroblasts, Fig. 5B. The range of the Gaussian function, the maximum value minus the minimum, indicating the number of fibres aligned in the most common direction compared to the least common direction, was much lower in *Vps33b*^*fl/fl*^*ER*^*T2*^ and *Vipas39*^*fl/fl*^*ER*^*T2*^ fibroblasts, Fig. 5C. This indicates fibres were more dispersed than in control cultures, suggesting changes in basement membrane deposition *in vitro*, consistent with defects in LH3 delivery causing alterations in collagen modifications and crosslinking.

Significant abnormalities in fibroblast deposition of collagen *in vitro*, Fig. 5A–C, yet subtle defects in collagen structure in Vps33b and Vipar deficient mice, Fig. 4, may indicate the effect of Vps33b and Vipar deficiency on LH3 function is not the primary cause of the scaly skin phenotype in *Vps33b*^*fl/fl*^*ER*^*T2*^ and *Vipas39*^*fl/fl*^*ER*^*T2*^ mice.

Co-cultures of primary fibroblasts and keratinocytes in a ‘full thickness’ skin model demonstrated that the defect is primarily epidermal. Control full thickness skin models stained for the lamellar body cargo kallkrein 5, KLK5, showed strong punctate staining in suprabasal keratinocytes, Fig. 5D. Whereas, KLK5 staining in knockout cultures was decreased in intensity and was less frequent. KLK5 staining was also decreased in intensity and frequency in control fibroblast-knockout keratinocyte cultures and knockout fibroblast-control keratinocyte cultures. This suggests the presence of control fibroblasts cannot rescue epidermal defects in knockout keratinocytes and the defects are likely to be due to an epidermal specific role of the VPS33B-VIPAR complex.

### 3.5 Vps33b and Vipar deficient mice display subtle defects in integrin function

VPS33B has been implicated in integrin endocytosis [20], to test whether integrin dependent functions were affected in Vps33b and Vipar deficient skin cells, we determined whether cell spreading and wound healing were altered. Cell spreading was not affected in VPS33B or VIPAR deficient primary keratinocytes or fibroblasts, Supplementary Fig. 2A–D. Wound healing was slower in *Vps33b*^*fl/fl*^*ER*^*T2*^ primary fibroblasts, Supplementary Fig. 2E and F, which may indicate subtle but significant defects in integrin recycling in *Vps33b*^*fl/fl*^*ER*^*T2*^ epidermis.

### 3.6 Vps33b and Vipar deficient mice have abnormal LB structures, altered LB cargo expression and decreased SC lipids

The main barrier to water loss in the skin is the SC, corneocytes surrounded by arrangements of lipid layers [40]. The water barrier defect in Vps33b and Vipar deficient mice may be due to defective SC formation. Skin from patients with ARC syndrome and ARKID syndrome, and *Vps33b*^*fl/fl*^*ER*^*T2*^ murine models, show SC defects and abnormalities in LBs [7,10]. *Vps33b*^*fl/fl*^*ER*^*T2*^ and *Vipas39*^*fl/fl*^*ER*^*T2*^ mice have larger corneocytes and thinner cornified envelopes, which may indicate LB deficiencies as cornified envelope formation is controlled by LB contents, Fig. 2C–E.

**Fig. 6:**
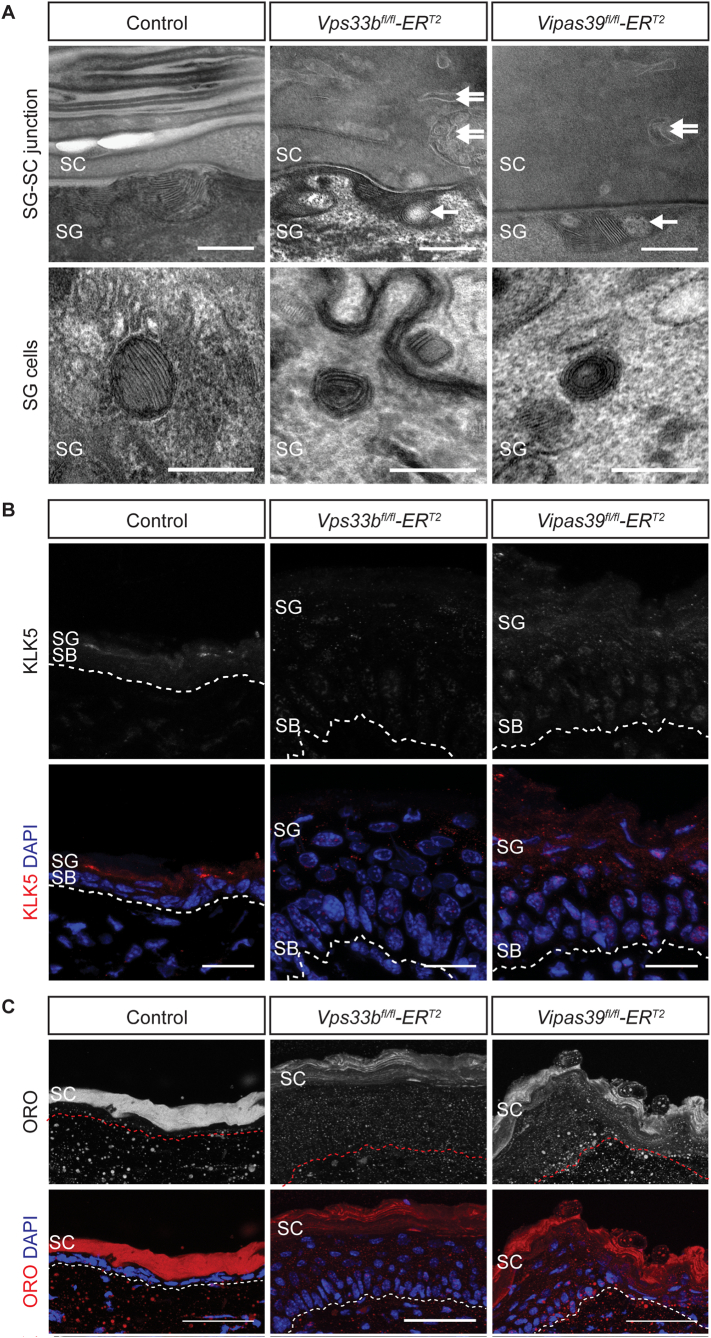
Vps33b and Vipar deficient murine epidermis shows defects in LB function. A – TEM images of the SG-SC junction in control, *Vps33b*^*fl/fl*^*ER*^*T2*^ and *Vipas39*^*fl/fl*^*ER*^*T2*^ epidermis. Bubble-like inclusion (single arrow), abnormal membrane structures (double arrow). Scale bars = 200 nm. Images are representative of at least two independent murine biopsies per genotype. B - TEM images of SG cells in control, *Vps33b*^*fl/fl*^*ER*^*T2*^ and *Vipas39*^*fl/fl*^*ER*^*T2*^ epidermis. Scale bars = 200 nm. Images are representative of at least two independent murine biopsies per genotype. C - KLK5 staining in control, *Vps33b*^*fl/fl*^*ER*^*T2*^ and *Vipas39*^*fl/fl*^*ER*^*T2*^ skin sections, counterstained with DAPI. All KLK5 images were taken with the same microscope settings, the brightness was boosted for the KLK5 DAPI merge to illustrate the localisation of KLK5 staining. Scale bars = 20 μm. Images are a maximum projection of a z-stack and are representative of results from at least six control and three *Vps33b*^*fl/fl*^*ER*^*T2*^ and *Vipas39*^*fl/fl*^*ER*^*T2*^ independent murine biopsies. D - ORO staining of control, *Vps33b*^*fl/fl*^*ER*^*T2*^ and *Vipas39*^*fl/fl*^*ER*^*T2*^ skin sections, counterstained with DAPI. Scale bars = 20 μm. Images are a maximum projection of a z-stack and are representative of results from at least six control and three *Vps33b*^*fl/fl*^*ER*^*T2*^ and *Vipas39*^*fl/fl*^*ER*^*T2*^ independent murine biopsies. SB, SG and SC layers are indicated.

LB ultrastructure is abnormal in *Vps33b*^*fl/fl*^*ER*^*T2*^ and *Vipas39*^*fl/fl*^*ER*^*T2*^ mice with bubble-like inclusions at the SG-SC interface, Fig. 6A. The LB cargo, KLK5, normally secreted by LBs, was present in discrete puncta in the suprabasal layers of control epidermis, Fig. 6B. However, in *Vps33b*^*fl/fl*^*ER*^*T2*^ and *Vipas39*^*fl/fl*^*ER*^*T2*^ epidermis KLK5 staining was decreased in intensity and more dispersed throughout the epidermis. This suggests there is reduced expression of KLK5 in *Vps33b*^*fl/fl*^*ER*^*T2*^ and *Vipas39*^*fl/fl*^*ER*^*T2*^ epidermis, which may be linked to defects in delivery of KLK5 to LBs.

Lipid deposition in the SC by LB was also affected in Vps33b and Vipar deficient mice. In the SC of control epidermis there is strong homogeneous staining in the SC for Oil Red O (ORO), which stains neutral lipids, Fig. 6C. However, in the SC of *Vps33b*^*fl/fl*^*ER*^*T2*^ and *Vipas39*^*fl/fl*^*ER*^*T2*^ sections ORO staining was reduced and restricted to regions of the SC that appeared to be arranged linearly, Fig. 6C. The linearity may be due to the increase in size of the corneocytes that was noted in these mice, Fig. 2E. Decreased intensity of ORO staining in *Vps33b*^*fl/fl*^*ER*^*T2*^ and *Vipas39*^*fl/fl*^*ER*^*T2*^ SC suggests there is a reduction in lipids secreted by LBs, further supporting the conclusion there are LB biogenesis and/or secretion defects in *Vps33b*^*fl/fl*^*ER*^*T2*^ and *Vipas39*^*fl/fl*^*ER*^*T2*^ mice.

## 4 Discussion

### 4.1 Vps33b and Vipar deficient mice develop a skin phenotype similar to ARC syndrome patients

VPS33B and VIPAR deficiencies lead to range of human phenotypes that always include ichthyosis [3,7]. Here we demonstrate that both Vps33b and Vipar deficient mice develop a skin disease that is similar to patients with ARC syndrome, with keratinocyte hyperplasia and hypertrophy, hypergranulosis, hyperkeratosis and ultrastructural defects in the SC. This demonstrates that Vps33b and Vipar deficient mice are suitable models to better understand the molecular mechanisms underlying the pathology of ARC syndrome skin disease. We demonstrate several defects in Vps33b and Vipar deficient murine epidermis including in keratinocyte differentiation, lamellar body function, basement membrane structure and integrin dependent functions.

### 4.2 Vps33b and Vipar deficient mice have notable defects in keratinocyte differentiation

*Vps33b*^*fl/fl*^*ER*^*T2*^ and *Vipas39*^*fl/fl*^*ER*^*T2*^ murine epidermis demonstrated formation of all keratinocyte layers of the epidermis, Fig. 1D, however, the epidermis was increased in thickness, Fig. 1E, and the basally expressed keratin 14 was not downregulated in suprabasal cells, Fig. 2A. The SC is the culmination of keratinocyte differentiation and the increased size of corneocytes of the SC with decreased thickness of the cornified envelope, Fig. 2C-E, suggests defects in terminal differentiation. The SC is also a major component of the water barrier of the epidermis [41], and defects in the structure of the SC may explain the decreased water barrier function of *Vps33b*^*fl/fl*^*ER*^*T2*^ and *Vipas39*^*fl/fl*^*ER*^*T2*^ skin, Fig. 1C.

### 4.3 Vps33b and Vipar deficient mice have abnormal lamellar body formation

The secretion of LB contents is vital for both the formation and maintenance of the structure and function of the SC and the epidermal water barrier [42]. It has been previously suggested that LB biogenesis and secretion might be affected by VPS33B deficiency in patients with ARC and ARKID syndromes [7,10]. We show that in addition to SC defects in *Vps33b*^*fl/fl*^*ER*^*T2*^ and *Vipas39*^*fl/fl*^*ER*^*T2*^ murine epidermis, Fig. 2C-E, which may be caused by defects in LB function, LB ultrastructure is also altered in *Vps33b*^*fl/fl*^*ER*^*T2*^ and *Vipas39*^*fl/fl*^*ER*^*T2*^ epidermis, Fig. 6A, with bubble-like inclusions amongst the lipid layers. Decreased expression and altered localisation of the LB cargo protein KLK5, Fig. 6B, and reduced amounts of lipid in the SC, Fig. 6C, also indicate defects in LB biogenesis. This is unexpected in an epidermis with increased water loss, as a defective water barrier has been linked to increased lipid synthesis and lamellar body secretion to promote recovery of the barrier [43]. There is likely, therefore, to be a defect in lipid synthesis or secretion in Vps33b and Vipar deficient murine skin.

The roles of VPS33B and VIPAR in LB function may be direct, in LB formation, delivery of LB contents to LBs, LB secretion or may be indirect. There was no accumulation of LB in the SG of *Vps33b*^*fl/fl*^*ER*^*T2*^ and *Vipas39*^*fl/fl*^*ER*^*T2*^ epidermis, which suggests there is not a defect in LB secretion, although this cannot be completely ruled out. Roles for VPS33B and VIPAR beyond LB biogenesis have been identified in several systems [5,21,22], therefore, the skin phenotype in VPS33B and VIPAR deficiency may also be affected by defects in other VPS33B-VIPAR dependent pathways and not solely LB dysfunction.

### 4.4 Vps33b and Vipar deficient mice have subtle changes in basement membrane structure

VPS33B and VIPAR also have an important role in collagen homeostasis, ensuring LH3 delivery to intracellular collagen [21]. Defective LH3 delivery and collagen homeostasis in patients with ARKID syndrome [7], suggested that defects in collagen homeostasis, such as in the basement membrane of the skin, could be affecting epidermal homeostasis.

Analyses of the overall organisation of the dermis, Fig. 4A, and collagen I fibres in the dermis, Fig. 4B, did not show defects in the overall organisation of these structures *in vivo*. However, basement membrane continuity was disrupted at the dermo-epidermal junction, Fig. 4C, consistent with alterations in LH3 delivery to collagen. Collagen I secreted by *Vps33b*^*fl/fl*^*ER*^*T2*^ and *Vipas39*^*fl/fl*^*ER*^*T2*^ dermal fibroblasts was also deposited in a less organised matrix, Fig. 5A–C, indicating that Vps33b or Vipar deficiency does affect dermal fibroblasts function.

The differences in matrix organisation between *in vitro* fibroblast cultures and dermal sections of Vps33b and Vipar deficient mice may indicate that dermal collagen morphology in *Vps33b*^*fl/fl*^*ER*^*T2*^ and *Vipas39*^*fl/fl*^*ER*^*T2*^ mice are not substantially affected by the knockout of Vps33b or Vipar post-development. These structures may not have turned over in the 4–5 weeks after induction of the knockout; corneocyte formation has been estimated to take 12 days after keratinocytes leave the stratum basale [44], whereas, collagen VII half-life in the dermo-epidermal junction has been reported to be about 30 days [45]. This could indicate epidermal defects in Vps33b and Vipar deficiency may present before defects in dermal structures and suggests that although VPS33B and VIPAR are important for collagen homeostasis through delivery of LH3 to collagen [21], changes in dermal collagen may not be the primary defect leading to the epidermal phenotype in Vps33b and Vipar deficient mice. This is also supported by the inability of control fibroblasts to rescue epidermal defects in KLK5 localisation and expression in co-culture with fibroblasts from Vps33b and Vipar deficient mice.

### 4.5 Vps33b and Vipar deficient fibroblasts have subtle changes in integrin function

VPS33B is reported to interact with β-integrins for endocytosis and integrin function [20]. In our Vps33b and Vipar deficient mice we assessed integrin dependent functions of cell spreading and wound healing. Cell spreading was unaffected by Vps33b and Vipar deficiency and wound healing was only significantly affected in *Vps33b*^*fl/fl*^*ER*^*T2*^ fibroblasts, indicating that there may be subtle but potentially important alterations in integrin traffic that may affect epidermal function.

### 4.6 Vps33b and Vipar deficient mice do not show junctional defects

VPS33B and VIPAR affect junction formation in some polarised cell types, including kidney cell lines [2] and hepatocytes [22], therefore it could be hypothesized defects in epidermal junctions in *Vps33b*^*fl/fl*^*ER*^*T2*^ and *Vipas39*^*fl/fl*^*ER*^*T2*^ mice would affect epidermal homeostasis and water barrier function. However, epidermal junctions were unaffected in *Vps33b*^*fl/fl*^*ER*^*T2*^ and *Vipas39*^*fl/fl*^*ER*^*T2*^ mice; tight junction and adherens junction proteins localised to the plasma membrane, Fig. 3A and B, tight junction function, as a barrier to ion movement, was not affected in primary keratinocyte cultures, Fig. 3C and D, and desmosome structure appeared normal, Fig. 3E. This is consistent with a role for VPS33B and VIPAR in LB and SC function which is not caused by changes in epidermal junctions.

## 5 Conclusions

VPS33B and VIPAR deficiencies lead to severe phenotypes in humans, in ARC and ARKID syndrome [3,7], and in mice, with constitutive knockout of Vps33b causing embryonic lethality [19,21]. This suggests essential functions for VPS33B and VIPAR, and, due to their ubiquitous expression, perhaps a ubiquitous function. However, diverse roles have been reported for VPS33B and VIPAR including megakaryocyte α-granule formation [5,19], platelet integrin recycling [20], LH3 delivery to collagen in kidney cells and fibroblasts [21], Rab11a-dependent apical protein targeting in hepatocytes [2,22], exosome maturation [23] and LB formation in the epidermis [7,10]. This indicates that VPS33B and VIPAR do have essential functions in mammalian biology, but their roles may be cell-type specific; ensuring post-Golgi protein delivery to diverse organelles.

In this study, we add to our knowledge of the pathology of Vps33b and Vipar deficient mice, demonstrating a range of abnormalities in Vps33b and Vipar deficient murine skin. Keratinocyte terminal differentiation was visible in *Vps33b*^*fl/fl*^*ER*^*T2*^ and *Vipas39*^*fl/fl*^*ER*^*T2*^ epidermis but the formation of the SC layer was altered, LB structure and localisation of a LB cargo was affected, the continuity of the basement membrane at the dermo-epidermal junction was disrupted, isolated fibroblasts secreted more disorganized collagen matrices, and *Vps33b*^*fl/fl*^*ER*^*T2*^ fibroblasts migrated slower in wound healing assays. The subtle changes in collagen structure and integrin function may affect epidermal function and keratinocyte differentiation, however, gross collagen fibril structure in the dermis was unaffected by the depletion of the Vps33b or Vipar and control fibroblasts did not rescue epidermal phenotypes of knockout keratinocytes, indicating that these defects may not be a primary cause of the epidermal phenotypes in Vps33b and Vipar deficient mice and suggests an epidermis-specific, and potentially LB formation-specific, role for VPS33B and VIPAR. A potential role for VPS33B in cytokine production was not explored but may be important in further study of the Vps33b deficient skin phenotype as elevated cytokine levels can affect keratinocyte fates [46,47].

Few trafficking proteins have been convincingly linked to the biogenesis and secretion of LBs; some evidence suggests LBs are formed from the TGN and form a tubulo-reticular cisternal membrane system towards the apical surface of keratinocytes, that may be connected to the apical plasma membrane [48]. However, there is also evidence to suggest that SNARE-mediated vesicular fusion events are required for correct LB structure and secretion, as a vesicular fusion protein SNAP29 has been implicated in this process. Epidermal deficiency of SNAP29 also causes hyperproliferation with acanthosis and hyperkeratosis, defects in epidermal differentiation, abnormal formation of LBs and reduced LB contents at the SG-SC junction [49]. Rab11a is also associated with LBs in the epidermis [50], is essential for LB biogenesis [51], and interacts with VPS33B and VIPAR [2]. This may indicate a common pathway for VPS33B, VIPAR, Rab11a and SNAP29 in epidermal homeostasis and LB formation.

VPS33B and VIPAR deficiency affects epidermal homeostasis and water barrier function in murine models. Alterations in epidermal junctions and gross changes in collagen morphology in the dermis were not evident in *Vps33b*^*fl/fl*^*ER*^*T2*^ and *Vipas39*^*fl/fl*^*ER*^*T2*^ epidermis, but LB formation and production of the lipid lamellae of the SC were affected. This indicates an epidermal-specific role for the VPS33B-VIPAR containing tethering complex. Whether this complex is required for LB formation, delivery of cargo to LBs or LB fusion at the plasma membrane remains to be determined. VPS33B and VIPAR have various reported roles in distinct cell types [2,5,[19], [20], [21], [22]] and we describe a further cell-type specific role in the function of epidermal LBs.

## Funding

Funding sources were not involved in study design; collection, analysis and interpretation of data; writing the report; or the decision to submit the article.

## Transparency document

Transparency document Image 1

## Appendix A Supplementary data

Supplementary figures 1–2 Image 2
