# Supplementary material for: VPS33B and VIPAR are essential for epidermal lamellar body biogenesis and function
Source: Biochim Biophys Acta. 2018 May;1864(5Part A):1609–21. doi: 10.1016/j.bbadis.2018.01.028 (PMC5906731; doi:10.1016/j.bbadis.2018.01.028)
Supplement: Supplementary file 2 — Supplementary figures 1–2 [file mmc2.pdf]

VPS33B and VIPAR are essential for epidermal lamellar body biogenesis and function

Clare Rogerson [a, b, +] and **Paul** Gissen [a, b, c]

[a] MRC Laboratory for Molecular Cell Biology, University College London, London WC1E 6BT, UK

[b] Institute of Child Health, University College London, London WC1N 1EH, UK

[c] Inherited Metabolic Diseases Unit, Great Ormond Street Hospital, London WC1N 3JH, UK

+ *Present address: Centre for Cell Biology and Cutaneous Research, Blizard Institute, Barts and The London School of Medicine and Dentistry, Queen Mary University of London, London E1 2AT*

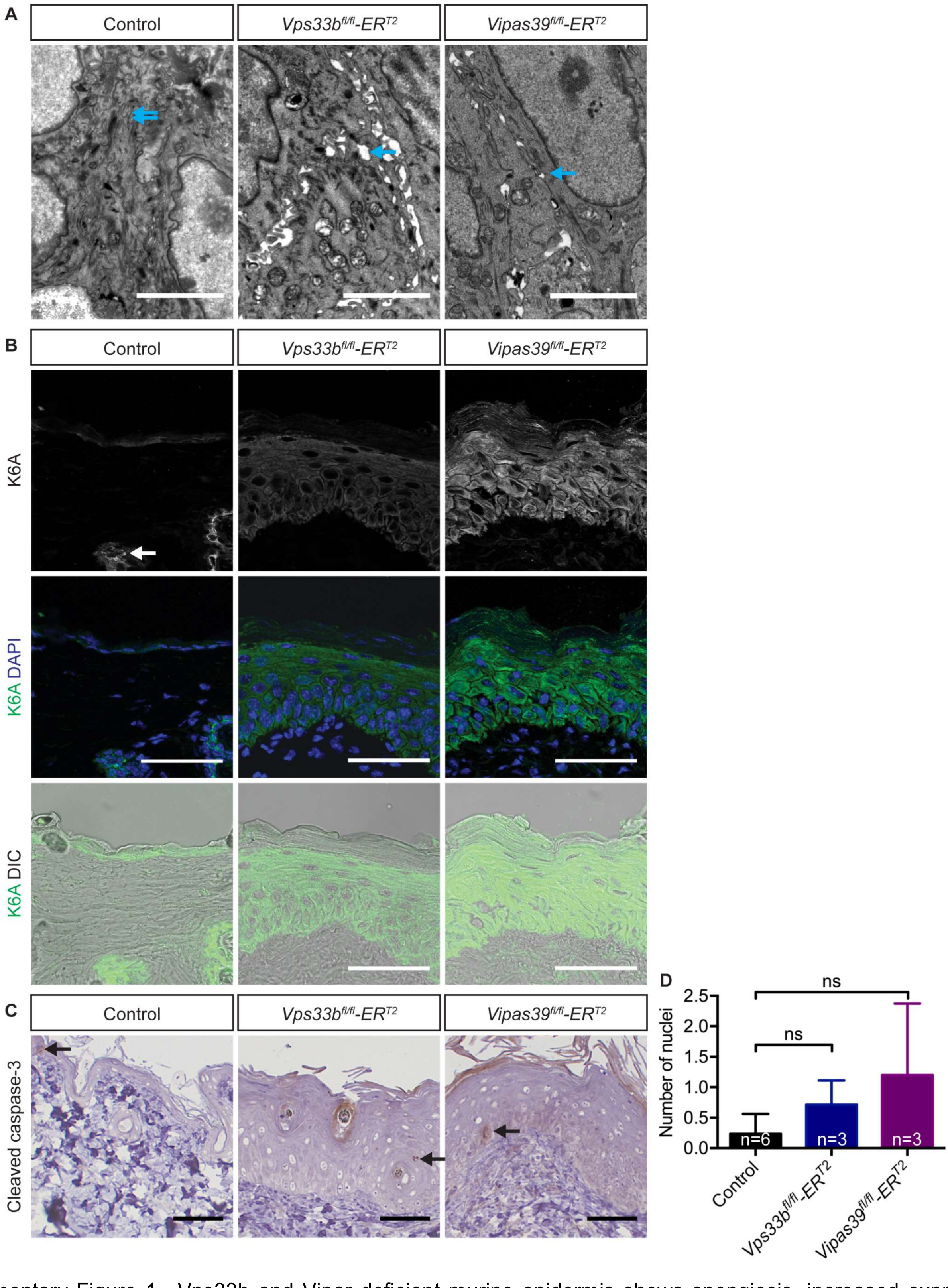

Supplementary Figure 1– Vps33b and Vipar deficient murine epidermis shows spongiosis, increased expression of hyperproliferative keratin K6A and no change in the expression of cleaved caspase-3 apoptotic marker. A - TEM images of control, *Vps33b$^{fl/fl}$ER^{T2}* and *Vipas39$^{fl/fl}$ER^{T2}* murine biopsies. Normal cell-cell contact (double arrow), wider gaps (single arrow). Scale bars = 5 µm. Images are representative of two independent mouse biopsies. B - K6A staining of control, *Vps33b$^{fl/fl}$ER^{T2}* and *Vipas39$^{fl/fl}$ER^{T2}* skin sections, counterstained with DAPI. Hair follicles (white arrow). Scale bars = 50 µm. Images are a maximum projection of a z-stack. DIC images are a single z-slice image. Images are representative of results from at least six control and three *Vps33b$^{fl/fl}$ER^{T2}* and *Vipas39$^{fl/fl}$ER^{T2}* independent murine biopsies.C – Immunohistochemical staining for cleaved caspase-3 in control, *Vps33b$^{fl/fl}$ER^{T2}* and *Vipas39$^{fl/fl}$ER^{T2}* epidermis. Cleaved caspase-3 positive cells (black arrow). Scale bars = 100 µm. Images are representative of results from at least six control and three *Vps33b$^{fl/fl}$ER^{T2}* and *Vipas39$^{fl/fl}$ER^{T2}* independent murine biopsies. D - Number of cleaved caspase-3 stained nuclei per mm of epidermis. Three fields of view analysed per mouse, number of mice analysed are indicated. Data are mean ± standard deviation, ordinary one-way ANOVA, non-significant (ns) $p>0.05$.

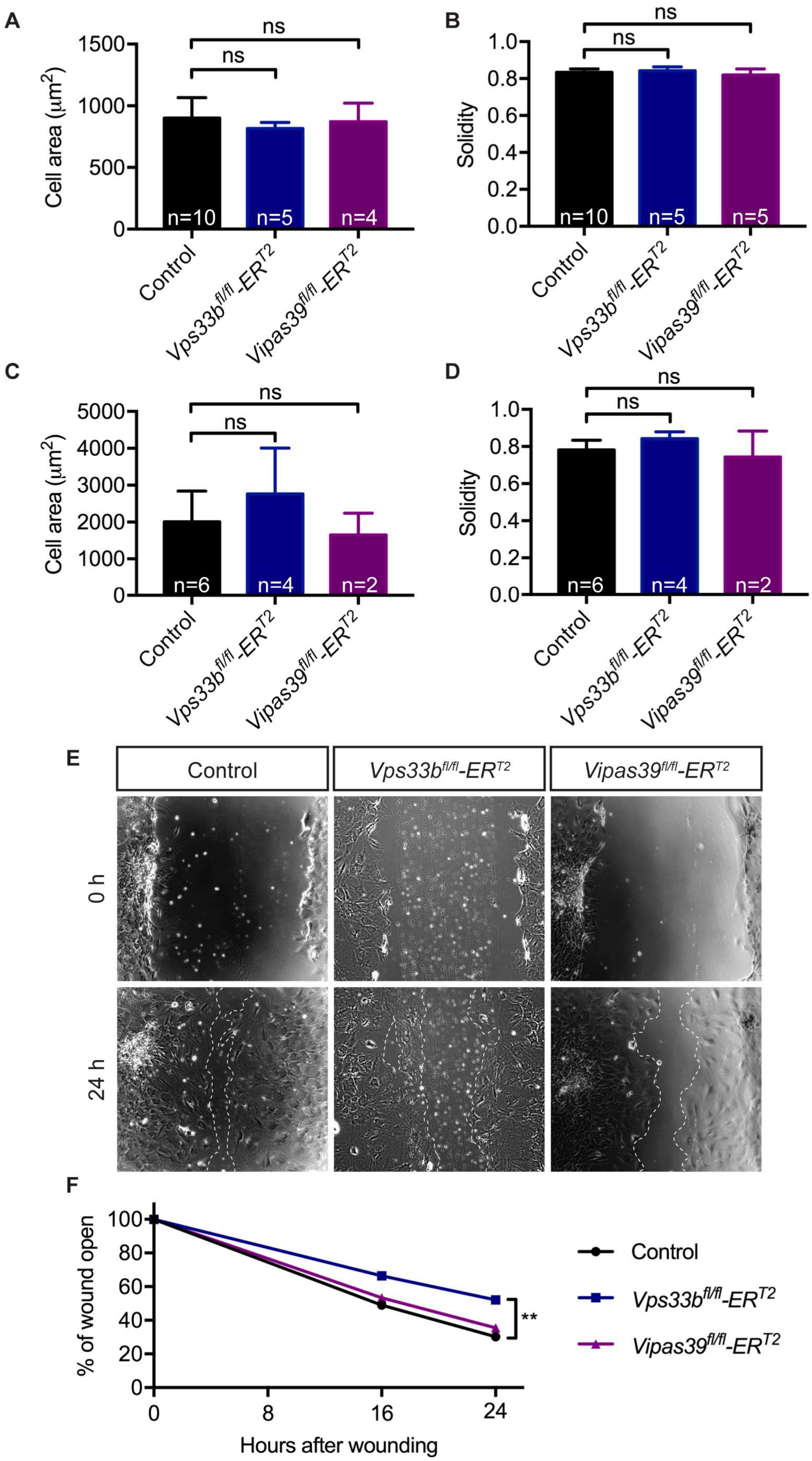

Supplementary Figure 2 - Cell spreading and wound healing are not substantially affected in Vps33b and Vipar deficient mice. A - Keratinocyte area after 1 h on fibronectin. B - Keratinocyte solidity after 1 h on fibronectin. C - Fibroblast area after 1 h. D - Fibroblast solidity after 1 h. For cell spreading at least three fields of view were measured per isolation, number of isolations analysed (n) are indicated in three independent keratinocyte and two independent fibroblast experiments. E - Representative images of wound healing experiments of control, $Vps33b^{fl/fl}ER^{T2}$ and $Vipas39^{fl/fl}ER^{T2}$ fibroblasts. F - Percentage wound closure in control, $Vps33b^{fl/fl}ER^{T2}$ and $Vipas39^{fl/fl}ER^{T2}$ wound healing assays. Results are representative of experiments from at least three independent fibroblast isolations. Data are mean ± standard deviation, ordinary one-way ANOVA, * $p \leq 0.05$, ** $p \leq 0.01$, all other comparisons were non-significant (ns) $p > 0.05$.
